# Social Capital, Deprivation and Psychological Well-Being among Young Adolescents: A Multilevel Study from England and Wales

**DOI:** 10.3390/ijerph17103420

**Published:** 2020-05-14

**Authors:** Kenisha Russell Jonsson, Joan Busfield, Marita Södergren, Miia Karen, Nicholas Kofi Adjei

**Affiliations:** 1 Department of Sociology, University of Essex, Colchester CO4 3SQ, UK; busfj@essex.ac.uk; 2 Department of Neurobiology, Care Sciences and Society, Karolinska Institutet, Alfred Nobels alle 23, 14183 Huddinge, Sweden; marita.sodergren@ki.se; 3 Unaffiliated Researcher, 16847 Broma, Sweden; miia.karen81@gmail.com; 4 Leibniz Institute for Prevention Research & Epidemiology-BIPS, Achterstrasse 30, 28359 Bremen, Germany; 5 Health Sciences Bremen, University of Bremen, Bibliothekstrasse 1, 28359 Bremen, Germany

**Keywords:** life satisfaction, mental health, social capital, socioeconomic deprivation, children/adolescents, neighbourhood

## Abstract

Examining the mechanisms influencing mental health and life satisfaction simultaneously allows for a better understanding of adolescents psychological well-being. Six indicators of neighbourhood social capital (NSC), neighbourhood socioeconomic deprivation (SecD) and their association with psychological well-being among young adolescents aged 10-15 from England and Wales were investigated. Using a random sample of 5201 adolescents (7253 observations) from the UK Household Longitudinal Study merged to aggregated local area census measures, we fitted a series of multilevel models. The findings showed that not being worried about crime and friendship networks mitigated the negative effects of deprivation on adolescent’s psychological well-being. These findings suggest that some forms of NSC may have a buffering and protective function, with the strongest effects in deprived neighbourhoods. We further found that psychological well-being of adolescents is dependent on both individual vulnerabilities and neighbourhood context. However caution is required if, and when public health policies are formulated to address this issue, given significant variations (27-36%) in the inter- and intra-individual psychological well-being were found among this group over time. Thus, policies designed to improve psychological well-being among adolescents should take into account the role of social processes in transmitting deprivation’s effects, as well as the various forms of social capital.

## 1. Introduction

Psychological wellbeing, defined as mental health and life satisfaction in this study, is an integral measure of public health, given the long-term and potentially crippling prolonged consequences of having poor psychological well-being [1]. Poor mental health is a leading cause of ill health, and it constitutes a major part of the global disease burden [2]. Growing numbers of adolescents— approximately 20% worldwide [3] and 10% in the UK [4,5]—suffer from various forms of mental health problems. At the same time, overall life satisfaction among this group is approximately 75% [3]. Although these measures are understood as not simply being on the opposite ends of the same spectrum, the literature lacks analyses exploring the mechanisms influencing these separate but interrelated measures simultaneously. The current study is aimed at bridging the gaps in the scholarship by examining the association between neighbourhood social capital (NSC) and neighbourhood socioeconomic deprivation (SecD) on the psychological well-being of young adolescents. The following sections briefly review the relevant literature and present the motivation and conceptual foundations for this current study.

### 1.1. Psychological Well-Being: Mental Health and Life Satisfaction

The impact of poor mental health is exemplified by the fact that self-harm and depression are among the leading causes of death and disability [6]. Moreover, there is evidence that the onset of common mental disorders (including self-harm, personality disorders, and attention-deficit disorders) usually begin at an early age and persist well into later life [7]. In cases where poor mental health begin at an early age, they impose significant social and economic costs on families of the affected youths and society at large [1]. Therefore, understanding the determinants of poor mental health and its impact on young adolescents is vital for understanding their psychopathological development and successful transition to adulthood.

Furthermore, having better knowledge of the factors that protect well-being can enable individuals overcome their mental health problems, which could in turn influence public policy, and allow early and effective interventions [8,9]. For example, studies that offer a more balanced assessment of psychological well-being may make important contributions to the growing literature on resilience; that is, the ability of individuals to overcome adversity and risk. Life satisfaction is a suitable measure given that it is a predictor of poor mental health [10], and it is able to capture both negative and positive aspects of an individual’s sense of well-being, life experiences, and their ability to function psychologically [11].

### 1.2. Social Capital and Socioeconomic Deprivation

Social capital (SC), a concept used to describe several interrelated and overlapping phenomena, is a well-known and widely employed measure in the assessment of health. It is associated with an individuals’ relationship to available resources and communal attributes such as trust, reciprocity, collective action, organizational participation and social networks [12,13]. Although there is still some debate surrounding the definition of SC, due to multiple operationalizations of the term, there is wide ranging consensus that the combination of “who you know”, “what you know” and “where you live” is intricately connected to, and perhaps influences health [14].

Based on this understanding of the term, two forms of SC commonly cited in the literature are bonding and bridging. Bonding and bridging SC allow an individual to obtain social support and mobilize solidarity [15] which, in turn, may influence overall health and well-being [16,17]. Bonding is often defined in terms of the relationships between individuals who share similar characteristics, homogeneity and strong norms; examples of which include relationships between family members, friends, and other close-knit groups. Bridging, on the other hand, is often applied to more heterogeneous group relationships. It is commonly measured using indicators of civic engagement and/or trust, and cooperation between groups of dissimilar status. Relationships associated with bridging social capital include those between neighbours or members of voluntary, political or sporting organisations.

The proposed mechanisms through which SC operates encompass physiological, behavioural and psychosocial explanations [18], as well as network theories, emphasizing the diffusion of ideas through a contagion model [19]. Despite the lack of consensus regarding the definition and the exact mechanisms through which SC operate, there is substantial empirical evidence that SC is an environmental and social resource that may influence the health of young adolescents through its interaction with wider social, political, economic and environmental determinants of health. In other words, the strength and direction of NSC may vary according to specific components of the measure tested [14,18]. Nonetheless, the overwhelming evidence is that SC may protect and buffer health from the negative effects of the neighbourhood.

Several studies suggest that SecD has a strong association with a number of factors shown to directly and indirectly influence the psychological well-being of adolescents such as perceptions of safety, area-level crime, area-level physical and social disorder, as well as concentrated and cumulative economic deprivation [20]. In particular, SecD is linked to increased incidences of, and/ or elevated levels of depression [21], psychological distress [22], emotional symptoms, hyperactivity, and conduct problems [23,24], and overall mental health difficulties [25] among young adolescents. Identical SecD may also affect the health of adolescents differently depending on individual circumstances, as well as, the amount of social capital to which they are exposed [26,27,28,29]. For instance, although ethnic minority youths are more likely to reside in economically deprived neighbourhoods, there is evidence that there is a racial/ethnic disparity in psychological well-being to their advantage [25]. This may be explained by the fact that, neighbourhoods with a high proportion of individuals from the same ethnic minority group are found to be high in certain forms of SC [30,31]. Indeed, prior studies found that NSC mediated the negative effect of socioeconomic deprivation; after adjustment for individual, family and neighbourhood characteristics [16,28].

### 1.3. Gaps in the Literature

The interplay between NSC and SecD remain under-researched, although they relate to the psychological well-being of young adolescents in the British context [29]. To date, the majority of scholarly work on this subject has been conducted in the United States (US) or elsewhere [27,32,33]. Whilst these prior studies offer important insights, differences in findings may be due to a number of reasons related to service provision, including the direct and indirect costs and general access to health care. The findings from studies conducted outside the UK may also lack geographic, socio-economic, cultural and demographic relevance. Previous studies have shown that the association between some aspects of NSC in the US appears to differ from that of the UK with regards to factors such as the social and demographic make-up of neighbourhoods. In the US, it has widely been argued that the diversity of a neighbourhood erodes social cohesion [13]. In contrast, evidence of these effects in the UK has been refuted by a number of studies [31,34,35]. Therefore, using British data means it is less likely to misattribute the findings from other contexts.

Research on the impact of NSC on adult health dominates the literature when compared to young adolescents. One explanation is the lack of large and robust national datasets with relevant area-level measures. Neighbourhood context matters for adolescents because at this critical stage of their cognitive and physical development, they are less mobile and autonomous compared to adults [36]. Therefore, spending a disproportionate amount of time within these localized environments could mean that social interactions and processes, and institutional resources may have a stronger (positive or negative) impact on their health and development. For this reason, studies exploring specific aspects of NSC influence on adolescents’ well-being is warranted, as reliance on adult-based findings could lead to misleading conclusions.

### 1.4. Conceptual Model

**Figure 1:**
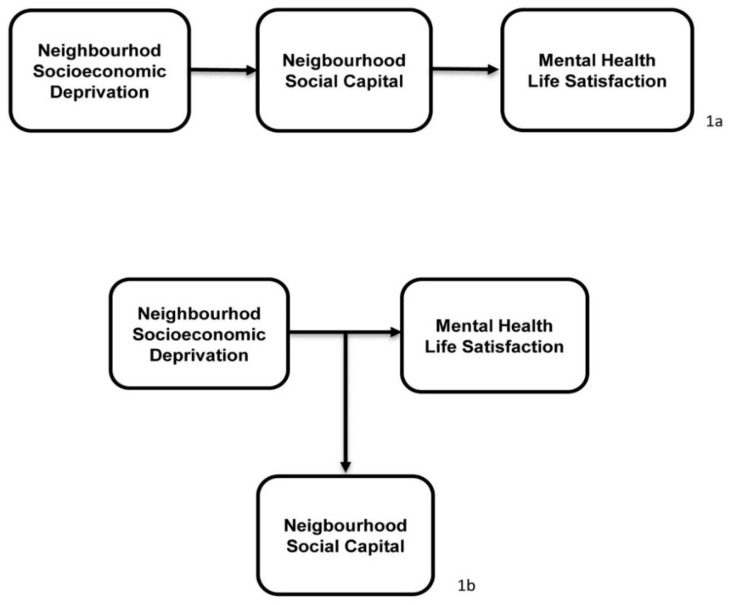
Conceptual models of the influence of social capital as a mediator (**a**) and moderator (**b**) of the relationship between socioeconomic deprivation, mental health and life satisfaction of young adolescents aged 10–15.

The conceptual model presented in Figure 1a, indicates that NSC mediates the psychological effect of residing in a SecD neighbourhood. Mediation is implied, if the magnitude or sign of the variable of interest increases or decreases when additional predictors are included in the model. Thus, by considering successive models with a different number of predictors, it is possible to evaluate the extent to which NSC explains the variation in the psychological well-being of adolescents. Research suggests further that the influence of any given set of socioeconomic conditions on an adolescent’s health may depend on their individual circumstances and the amount of SC available to them [27,28,29]. In other words, the level of resilience displayed vary in different risk contexts [37].

What these studies allude to is the relationship presented in Figure 1b. It states that NSC acts to moderate the influence of residing in a SecD neighbourhood on the psychological well-being of young adolescents. According to this, the impact of NSC is unevenly distributed. Take, for example, the study by [27], which found that the interaction between SC and higher levels of SecD was associated with lower perceived health. Another study on indicators of mental health suggested that the incidence of internalising problems among adolescents decreased as the size and quality of their social networks increased, but the magnitude of this effect varied with SecD [28]. However, findings relating to SC as a moderator is mixed. Whilst some studies found that in the context of severe disadvantage protective factors are less effective [26,38], others suggest that they are more beneficial for individuals living in high risk environments [9]. These findings point to the need for greater clarity on whether protective factors work similarly across all levels of neighbourhood disadvantage or whether their benefits are limited to specific contexts. In this study, moderation models were used to examine the associations, between NSC and psychological well-being among young adolescents, and whether it varies based on residency in a neighbourhood with greater or lesser level of SecD.

### 1.5. Study Aim

Based on the above conceptual model, we considered the following questions: To what extent does NSC mediate and/or moderate the relationship between SecD and the psychological well-being of young adolescents? Does NSC explain psychological well-being after adjustment for neighbourhood, individual and parental predictors of health?

To obtain robust and generalizable conclusions, the analysis was conducted using two large national datasets; UK Household Longitudinal Study (UKHLS), based on individual-level repeated measures that contains information on adolescents and their families, linked to aggregated geocoded data from the 2011 UK census. This work fills some important knowledge gaps because the literature lacks studies: (i) affecting young adolescents; (ii) in European contexts— in particular studies from Britain; (iii) relating to the inclusion of life satisfaction as an outcome; and (iv) based on large and robust nationally representative data.

## 2. Materials and Methods

### 2.1. Data

Data for this analysis were drawn from multiple sources. Individual-level repeated measures from waves 1, 3, and 5 of the UKHLS [39]. This is an annual longitudinal household panel survey that started in 2009, with a nationally representative and stratified cluster sample of approximately 40,000 households in the United Kingdom. The sample for this study consisted of young adolescents aged 10-15 of adult panel members, for whom parental consent to participate was granted, and who responded to the questionnaire [40]. The second source of data consists of aggregated geo-coded administrative measures—so called neighbourhood measures—collected in the 2011 UK census [41]. It is derived from census-defined small area statistics, at the middle super output area (MSOA) level, have a minimum residential size of 5000 individuals, 3000 households and an average population size of 7500. The use of MSOAs made it possible to link these aggregated area-level measures taken from the 2011 census to the individual responses gathered in the UKHLS.

### 2.2. Sample

Changes in the survey sample arose due to casewise deletions of some sample members with missing information on relevant variables, attrition, and the inclusion of new participants in the survey. Attrition may have occurred across the waves because: (a) the survey team lost contact with a family who participated in an earlier wave, (b) a young person decided to stop responding to the survey, or (c) an individual initially classified as a youth (aged 10–15) joined the adult panel upon turning 16. Sample changes also occurred when younger children became eligible for inclusion upon reaching the age of 10 and thus entered the youth panel, and when children of an appropriate age joined households participating in the survey.

### 2.3. Missing Values

Analysis of missing values identified three major sources of missing values. Neighbourhood identifiers necessary to merge the individual and neighbourhood information was missing for 211 MSOAs (7% of the initial sample). This meant that it was not possible to calculate the requisite measures for those sample members; as such, they were dropped from the analysis. Approximately 15% of the initially sampled children did not disclose their ethnicity. Finally, information on length of residence which affected 9.5% of the original sample (used as a control variable in this study) was unavailable for young adolescents incorporated into the UKHLS during wave 2. This information appeared to be missing because the UKHLS only asked respondents not previously interviewed, and participants drawn from an earlier survey were considered to have previously been interviewed. Despite these missing values, an examination of the complete versus missing cases, indicated that the cases with missing values did not differ greatly from the original sample. Therefore, casewise deletion was used in preference to imputation because it was not expected to introduce appreciable bias. Results of the analysis of missing data is available upon request.

### 2.4. Final Sample

The final analytical sample for this study (obtained after deleting subjects with missing information) was an unbalanced panel consisting of 5201 (7253 observations) adolescents aged 10–15 (*M*_age_ = 12.5 years, *SD* = 1.70) clustered in 2393 neighbourhoods across England and Wales. The participants were almost equally distributed across six cohorts (1994–1999) and by gender (50.2% female). The sample was diverse in terms of ethnicity (72.8% White, 5.9% Mixed, 13.6% Asian, 7.1% Black, and 0.7% of other or multiple ethnicities).

### 2.5. Measurement of Outcome Variable

#### Psychological Well-Being 

Two dependent measures of psychological well-being were assessed, mental health and life satisfaction. *Mental health* based on responses to the self-reported version of the Strengths and Difficulties Questionnaire (SDQ) collected at waves 1, 3 and 5 of the UKHLS. The SDQ is a screening instrument validated for cross-national assessments [42] and well-used across multiple ethnicities [43]. The instrument consists of five subscales. Four indicating problem areas (emotional symptoms, conduct problems, hyperactivity-inattention, peer problems) and one area of strength (prosocial behaviour). Each subscale has five items with responses scored on a three-point Likert scale, coded 0 ‘not true’, 1 ‘somewhat true’ and 2 ‘certainly true. The sum of problem areas yields a total difficulties score, ranging from 0 to 40, with higher scores indicating poor mental health. *Life satisfaction* measured using a single item with seven pictorial representations corresponding to respondent’s feelings regarding their circumstances. As in previous studies, responses were reversed coded such that higher scores indicated greater life satisfaction [44]. This measure of life satisfaction has not been cross-culturally validated, but single-item measures of life satisfaction have been widely used and are accepted as indicators of overall well-being among both adults and adolescents [11,45].

### 2.6. Independent Variables

#### 2.6.1. Neighbourhood Social Capital (NSC)

Measured at wave 3 of the *UKHLS* survey and based on six separate indicators of parents’ perceptions of their neighbourhoods (Table 1). The measures represent the aggregate standardized mean responses within each neighbourhood, and were coded so that higher scores corresponded to higher levels of SC. Similar operationalizations of SC have been used in earlier studies [32,33].

#### 2.6.2. Socioeconomic Deprivation (SecD)

Measured using two indicators the *Townsend deprivation index* and the proportion of economically active individuals. The Townsend deprivation index is designed to capture the material conditions of a given area using four (household overcrowding, the percentage of unemployment, non-home and non-car ownership) census aggregated MSOA level measures [46]. Average deprivation as measured by the index was moderate (mean = 0.46, SD = 2.25) but varied widely across neighbourhoods, ranging from −2.56 to 9.22. The *proportion of economically active individuals* reflects a single (and temporary) aspect of a neighbourhood’s economic status. The measure was calculated by dividing the number of economically active individuals in the MSOA by its total number of residents [41]. The proportion of economically active residents in the studied neighbourhoods ranged from 0.32 to 0.88 (mean = 0.69, SD = 0.06).

#### 2.6.3. Parental Measures

These indicators, previously linked to adolescents’ mental health [22,25] and life satisfaction [45] were included in the models. These were averaged over the two parents, with the exception of education, for which an indicator based on the parent with the highest level of educational attainment was used. Across all waves, 92% of parental information related to mothers. *Lone parent household* is a dichotomous indicator of adolescents living in a single parent household. Pertained to 26.4% of the sample. *Household income.* The monthly total household net income with no deductions, divided into terciles. *Country of birth, **c***ategorized as (1) both parents born in the UK (78.3%), (2) one parent born outside the UK (10.8%), and (3) both parents born outside the UK (10.9%). *Length of residency.* A large plurality of sample members (44.8%) had resided in their neighbourhood for more than ten years, whilst approximately 43.2% resided in their neighbourhoods between four and ten years, 8.0% between two and three years and the remaining sample members (4.0%) for a year or less. *Highest level of education*, assessed by a single measure coded into six categories: (i) No qualifications, (ii) Other qualification, (iii) A- level (including Welsh baccalaureate, international baccalaureate; higher grade/advanced higher and certificate of sixth year studies) (iv) GCSE includes CSE standard/ordinary (O) grade/lower; (v) other higher degree; and (vi) degree. The most common level of parental educational attainment was degree (31.7%), followed by GCSE or similar (20.0%) and then A-level (19.9%). *Employment status,* measured using a single dichotomous indicator, which showed that the majority of young adolescents lived in households 82.4% where at least one parent worked. *Mental health,* the mean scores for both parents, based on a subset of the scores related to mental functioning from the well-used and validated 12-item short form questionnaire [47]. Scores ranged from 0 (low functioning) to 71.6 (high functioning), with a mean of 48.5 (*SD* = 9.2).

#### 2.6.4. Wave (Time of Data Collection) 

This was included in every model to control for and assess changes in outcomes over the studied calendar periods. The wave including the greatest proportion of participants was wave 1, which ran from 2009 to 2011 and included 46.2% of the study’s final sample. Wave 3 ran from 2011–2013 and included 29.6% of the sample; wave 5 ran from 2013–2015 and included 24.2% of the sample.

### 2.7. Statistical Analysis

To account for the hierarchical nature of the data, and to avoid underestimation of the standard errors while improving the precision of the estimates, three-level multilevel linear regression models were fitted using Stata 14 [48]. The equation for the simplest form of a random-intercept model is shown below:

(1) $$y_{ijk}=\beta_{0}+\beta_{1}X_{1ijk}+\beta_{2}X_{2j}+\beta_{3}X_{3k}+v_{k}+u_{jk}+e_{ijk}$$

where youth-waves *ijk* are nested in youths *jk* who are in turn nested in neighbourhoods *k*. $v_{k}$ and $u_{jk}$ are neighbourhood and individual random intercepts; like the individual-wave error term $e_{ijk}$, they are normally distributed with mean 0 and standard deviations $\sigma_{v}^{2}$, $\sigma_{u}^{2}$, and $\sigma_{e}^{2}$ respectively. An extended version of this model was analysed in which the random intercepts and slopes at both the neighbourhood and youth levels were allowed to covary so that the intercept and slopes could be correlated.

This model accounted for the fact that the data consisted of repeated measures (from waves 1, 3 and 5) of reported mental health and life satisfaction (level 1) for adolescents (level 2) clustered within neighbourhoods (level 3). Further, applying this type of multilevel model makes it possible to partition and explain the variation within-individuals over time, between individuals, and between neighbourhoods. Using a multilevel model also made it possible to account for the fact that the *UKHLS* sampled adolescents from the same MSOAs, and to control for the similarities between the studied neighbourhoods. An additional benefit is controlling for correlations arising from repeated responses for the same individual.

### 2.8. Model Description

The models assessed in this study were analysed sequentially and separately for mental health and life satisfaction (Table 2).

## 3. Results

Table 3 summarizes the basic descriptive statistics for the all measures used in the study for the total sample and across the three waves.

### Multilevel Models

Table 4 presents the multilevel regression coefficients and standard errors from successive models. The modelling strategy adopted to address the two outcomes (mental health and life satisfaction) was identical. In the description of the results that follows, the results of Models 1 and 2 are not shown in Table 4 but is available upon request. 

An assessment of the intra-class correlation (ICC)—a measure of dependencies in clustered data—indicated that variation among adolescents accounted for most of the observed variation in mental health and life satisfaction. The models assessing mental health yielded ICC values indicating that 62% of the stable variation was attributable to inter-individual variation, with only 11% of the total variation attributed to the neighbourhood. This variation remained unexplained because in later models, even after full adjustment for the individual and parental predictors and neighbourhood deprivation, most of the within-individual (61%) and between-neighbourhood (10%) variation remained. Assessments of the ICC values for models examining variation in life satisfaction similarly indicated that 12% and 52% of the variation in reported life satisfaction was attributable to neighbourhoods and adolescents, respectively. These results indicate that there was substantial intra-individual variation in life satisfaction over time. After including all other measures, the between-neighbourhood and adolescent-level variation declined to 10% and 51%, respectively. Conditional on the fixed-effects covariates, the results indicate that poor mental health and life satisfaction was weakly correlated within neighbourhoods but was highly correlated within individual adolescents.

Assessments of the ICC, indicated that a substantial proportion of the total variation (approximately 27% for mental health difficulties and 36% for life satisfaction) was not explained by these models. A logical assumption is that this unexplained variance consisted of a combination of inter- and intra-individual changes in psychological well-being among adolescents over time, and that it might be the result of normal fluctuations, measurement error, and/or random noise. Additionally, estimates for the random part of the model shows a negative correlation between the intercept and slope at both the youth and neighbourhood levels, suggesting that both adolescents and neighbourhoods with better mental health at the baseline tended to show the greatest deterioration over time. Similarly, an examination of the random part of the model yielded a negative covariance estimate at the adolescent level when results of models for life satisfaction. These indicate that the mean rate of change in life satisfaction was lowest among adolescents who initially reported above average life satisfaction.

The results for analyses related to mental health showed that Asian and Black youths had significantly better mental health when compared to their White counterparts. In addition, the coefficients indicated a negative association (i.e., better mental health) for young adolescents when one or both parents were non-UK-born, had high mental functioning, when at least one parent is employed, and had resided in the same neighbourhood for 10 years or more. However, the average reported mental health problems did not differ between data collection points. The results related to life satisfaction indicated that as adolescents got older, girls and youths from single-parent household had significantly lower life satisfaction.

In Model 3 the coefficients indicated that neighbourhood SecD was associated with reduced psychological well-being among adolescents. That is, young adolescents living in greater deprivation and with a high proportion of economically inactive residents were more likely to exhibit poor mental health and lower life satisfaction. The results indicated however, that deprivation measured by both the Townsend deprivation index and the proportion of economically active, was more consistently associated with life satisfaction when compared to the association of deprivation with mental health. This is shown by the coefficients related to deprivation, especially the proportion of economically active in the neighbourhood, which persists across every tested model. We found a positive association between poor mental health and being economically active. The high proportion of job seekers and underemployed individuals residing in these deprived neighbourhoods may explain this phenomenon.

Model 4 was used to examine whether NSC mediated the effects of deprivation on well-being. The results indicated however, that four of the measures of NSC (quality of facilities and amenities, civic engagement, trust and cooperative norms, and social cohesion) did not have a significant influence on the well-being of adolescents. In contrast, not being worried about crime and friendship networks mediated the impact of deprivation on poor mental health. This is illustrated by the fact that the small but significant relationship between deprivation and mental health disappeared once NSC was included in the model. Thus, pointing to the fact that NSC attenuates some of the negative effects of deprivation on the mental health. The results indicate similarly that NSC mediates some of the negative effects of deprivation on life satisfaction. Young adolescents living in neighbourhoods characterised by low worry about crime and friendship networks reported higher levels of life satisfaction.

**Figure 2:**
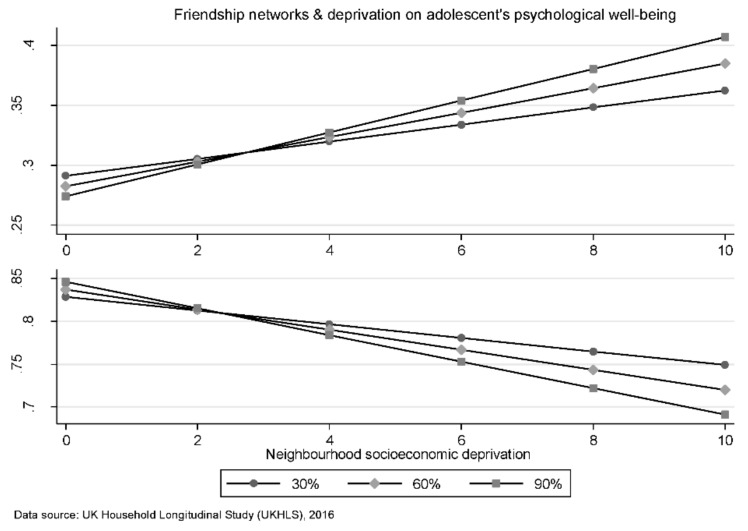
Estimated marginal mean effects (at representative values) of neighbourhood deprivation and friendship network on psychological well-being. X-axis higher numbers indicate greater socioeconomic deprivation, on the y-axis higher numbers indicate life satisfaction and mental health, and the lines represent the marginal effects at various levels of friendship networks (i.e., 30%, 60%, and 90%) within a given neighbourhood. Scales standardized.

When moderation effects (Model 5) are considered, the only measure of NSC that interacted with deprivation (as measured by the Townsend index) and was significantly associated with well-being, was neighbourhood friendship networks. The associations between SecD and psychological well-being of young adolescents’ vary by the strength of friendship networks across different neighbourhoods. Young adolescents residing in neighbourhoods with higher average friendship networks appear to enjoy better mental health and higher life satisfaction. This relationship is strongest in neighbourhoods of greater deprivation (see Figure 2).

## 4. Discussion

This study examined how several indicators of NSC are associated with the psychological well-being, measured as mental health and life satisfaction, of young adolescents aged 10–15 years old; and to test the consistency of findings across this population sub-group based on the level of deprivation within their neighbourhoods. Overall, the findings showed that some aspects of psychosocial and physical context in which young adolescents reside matters for their psychological well-being. These findings are similar to prior studies, which found that NSC plays a role in the transmission of the effects of neighbourhood deprivation on young adolescents’ well-being [7,8,41].

Together the mediation and moderation models indicated that some components of NSC both buffers and protects adolescents from more negative aspects of the neighbourhood in which they reside. For instance, statistically significant relationships were found between not being worried about crime and friendship networks and both measures of psychological well-being selected for this study. Thereby indicating that neighbourhoods high in social capital related to the psychosocial context may have a stronger influence on well-being when compared to the individual, and parental measures, as well as, the level of SecD within the neighbourhood. However, there were some inconsistencies in the patterns of association found. We found different relationships between the indicators of NSC, deprivation and the well-being measures. For instance, the pattern of the associations between NSC was similar for mental health and life satisfaction although they operate in opposite directions. In contrast, a wider range of individual and parental measures were associated with poor mental health, meanwhile, life satisfaction had a stronger and more consistent association with neighbourhood deprivation. This suggests that the psychological well-being of young adolescents is dependent on both individual vulnerabilities and the area-level context. Moreover, these findings suggest that the factors that are relevant for promoting good health and resilience are not necessarily the same as those necessary to prevent mental health difficulties. Hence, a greater number of studies are required to assess, the risk and resiliency factors that are integral for the healthy development of young people.

The above findings were further emphasized by the results of the interaction between NSC and deprivation which signalled that some components of NSC moderated the influence of the neighbourhood by reducing the socioeconomic disparity in adolescent psychological well-being. In particular, investing in neighbourhood friendship networks during adolescence is beneficial, irrespective of socioeconomic background, because it has a protective influence on psychological well-being.

It is also, important to note that the lack of significant associations between the psychological well-being measures and some indicators of NSC should not be interpreted as evidence of no effect. The results presented here may be an artefact arising from the fact that the focus of the study was young adolescents. Previous studies found non-linear age differences in the effects of the neighbourhood socioeconomic conditions and social processes on youth development [20]. These results may be an indication that neighbourhood influences may not be as strong during the stage of the life cycle assessed in this study. The results further indicate that a reliance on the evidence arising from adults may be misleading, as it does not provide a complete picture of the relationship between NSC and the health of adolescents.

Inconsistencies between our findings and previous studies may be partly due to variations in the measures of psychological well-being examined and/or the operationalization of this measure. Another explanation for the lack of consistency in results pertaining to NSC and its association with the psychological well-being of young adolescents may be that different components of NSC has been examined in this study when compared to prior studies. Some studies based the indicators of NSC on the adolescents’ experiences as active social agents in their own right [32] while others like this study used area level measures based on the adults’ experience of the neighbourhood. Finally, as proposed by [49] the differences in the results may be explained by the fact that different types of SC provide different types of support, and as such, leads to different health associations. For instance, past studies point to the particular role of the bonding and bridging components of social capital in relation to psychological well-being [32] and other health outcomes [50].

### Strengths and Limitations

The present study has several strengths and limitations that warrant discussion. A reason that research in this area has remained scant is a lack of relevant data. Few data sources include questions measuring NSC from the perspective of adolescents, giving rise to a gap in the literature that this study addresses. Despite this, an acknowledged weakness of this study is its reliance on parental perceptions and experiences of NSC rather than those of the adolescents themselves. While parental perceptions and experiences may differ from those of adolescents, it has been shown that parents’ SC influences their children’s SC and health [51,52]. This is because parental networks and characteristics influence the types of relationships and resources that their children can access. In fact, [53] suggested that parents’ perceptions may stimulate adolescents’ reactions to their neighbourhood and the associated coping strategies. Several studies suggest that parental characteristics enhance children’s well-being. For example, parental social support and monitoring are stronger in less deprived neighbourhoods when compared to more deprived neighbourhoods [5,44,45]. Furthermore, the use of a parental measure of SC rather than one based on the adolescents’ opinions offers a more independent evaluation of the neighbourhood. This reduces/removes the problem of endogeneity that might be present in studies based on self-assessment of health, which may be confounded with individuals’ experiences of their neighbourhoods. In addition, if SC is indeed a communal resource, as presented in the literature, it could be argued that the effects of NSC if not the same for young adolescents, it should be similar to that of their parents.

MSOAs has been widely used to delineate neighbourhood geographic boundaries in the UK. Given that, they are primarily defined for administrative purposes (to monitor social, economic, and general living conditions across different geographies), the boundaries of an MSOA may not coincide with neighbourhood boundaries as defined and experienced by people living within that MSOA. MSOA-level data may not fully characterize these neighbourhoods as experienced by people living within that MSOA. Using MSOA-level data, could lead to a mismatch between usage and definition, and this may influence the reliability of the evaluations. A focus on smaller geographic areas could potentially have yielded neighbourhood delineations that closely reflect the definitions of people living within the studied areas, and this might have resulted in the identification of stronger neighbourhood effects. MSOA-level data were ultimately used in preference to more granular alternatives to protect the participants’ anonymity.

The sample changed across the data collection periods due to attrition, missing values, and the inclusion of new panel members, all of which may have made the panel unbalanced. However, it was possible to account for the fact that some adolescents did not contribute to every data collection period because the chosen models can handle unbalanced panels [54]. These issues notwithstanding, the sample was drawn from multiple diverse communities across England and Wales, and was based on sampling procedures that captured a wide cross section of the British population. This increases the generalizability of the findings to communities that were not included in this assessment.

Adolescents aged 10-15 years currently make up approximately 7% of the population of England and Wales, and this number is expected to grow over the coming decade [55]. Therefore, despite the modest health benefits of SC, research into its possible implications for the health of the next generation is vital given the absolute number of lives that may be influenced by the extent and density of the SC in their area of residence.

## 5. Conclusions

The findings of this study emphasized from a public health and policy stand point the importance of considering the impact of both psychosocial and physical environments when examining their relationship with psychological well-being among adolescents. While the empirical evidence only partially supported the hypothesized models, the study has highlighted the importance of cultivating various forms of SC because these different components appear to offer differing benefits. For both public health and policy reasons, interventions seeking to improve adolescents’ mental health and life satisfaction must therefore both reduce socioeconomic disadvantage and enhance the psychosocial benefits that can be reaped from SC. This study not only demonstrates the need to consider when and how SC may positively influence psychological well-being among young adolescents, but also indicates a need for more research on the possible negative effects of some aspects of SC. Finally, it provides evidence that the effects of SC may be non-linear.

## Figures and Tables

**Table 1:** Definition and description of survey items, and the corresponding social capital indicators.

Variable Name	Items Included in Measure	Responses Provided in Survey	Range (Mean, SD) Cronbach’s Alpha
Worry about crime	Worry about being affected by crime	Worried about crime [0], Not worried [1]	0 to 1 (mean = 55, SD = 0.23)
Neighbourhood facilities	Standard of local services for primary schools, secondary schools,	Items coded from 1 [poor facilities] to 4 [excellent facilities]	−0.75 to 0.53 (mean = −0.01, SD = 0.14), α = 0.66
	medical services, shopping; leisure, local transport		
Friendship networks	Proportion of friends with similar age, race, level of education, income and reside in the local area	Items coded 1 [less than half] to 4 [all similar]	−0.1.83 to 1.28 (mean = −0.06, SD = 0.30), α = 0.58
Civic engagement	Are you currently a member of any of the kinds of organisations on this card? Sixteen (16) types of organisations listed including political, voluntary, professional, and recreational clubs	No [0], yes [1]	0 to 1 (mean = 0.51, SD = 0.23)
Trust and cooperative norms	Close-knit neighbourhood	Items (1,2 and 3) reverse coded. All items range from 1 [ strongly disagree] to 5 [strongly agree]	−0.71 to 0.73 (mean = 0.01, SD = 0.14), α = 0.90
	*People are: (1) willing to help their neighbours, (2) they can be trusted, (3) get along with each other*		
*Social cohesion*	*Belong to neighbourhood, Local friends mean a lot, able to obtain advice locally, can borrow things from neighbours, willing to improve neighbourhood, plan to stay in neighbourhood, I am similar to others in neighbourhood, Talk regularly to neighbours*	*Items coded from 1 [ strongly disagree] to 5 [strongly agree]*	*−0.38 to 0.44 (mean = 0, SD 0.07), α = 0.86*

Notes: Higher scores correspond to higher levels of social capital. Source: Understanding Society (2015) Wave 3 mainstage questionnaire linked with MSOA-level data from census 2011.

**Table 2:** Summary of multilevel regression models examined in this study.

Models	Specification
Model 1	Waves
Model 2	Model 1 + covariates ^a^
Model 3	Model 2 + socioeconomic deprivation ^b^
Model 4	Model 3 + social capital ^c^
Model 5	Model 4 + social capital * Townsend deprivation index

Notes: ^a^ Adolescents: cohort, gender, ethnicity; Parental measures: lone parent households; household income in tertiles; parents’ nativity; at least one parent in the household working; length of residency in the neighbourhood; parents’ highest education; parental mental health and takes into account area-level clustering. ^b^ Measured using the Townsend deprivation index and the proportion of economically active residents per MSOA. ^c^ Measured by not worried about crime; neighbourhood quality facilities & amenities; civic engagement; friendship networks; trust & cooperative norms and social cohesion. * Indicates an interaction between the two measures.

**Table 3:** Youth, parental and neighbourhood characteristics for the total sample and the sample at each wave.

Unweighted n (%)	Total (n = 7253)		Wave 1 (n = 3351)		Wave 3 (n = 2149)		Wave 5 (n = 1753)	
**Individual/Parental measures**								
Girl	3642	50.2	1693	50.5	1088	50.6	861	49.1
Cohorts								
1999	1141	15.7	542	16.2	342	15.9	257	14.7
1998	1181	16.3	576	17.2	333	15.5	272	15.5
1997	1212	16.7	541	16.1	367	17.1	304	17.3
1996	1236	17.0	584	17.4	361	16.8	291	16.6
1995	1257	17.3	552	16.5	380	17.7	325	18.5
1994	1226	16.9	556	16.6	366	17.0	304	17.3
Ethnicity								
White	5282	72.8	2389	71.3	1595	74.2	1298	74.0
Mixed	428	5.9	184	5.5	132	6.1	112	6.4
Asians	983	13.6	487	14.5	266	12.4	230	13.1
Blacks	512	7.1	271	8.1	136	6.3	105	6.0
All other ethnicity	48	0.7	20	0.6	20	0.9	8	0.5
Single parent household	1916	26.42	934	27.9	548	25.5	434	24.8
Parents Nativity								
Both parents UK born	5682	78.3	2602	77.7	1698	79.0	1382	78.8
1 parent non-UK born	781	10.8	340	10.2	252	11.7	189	10.8
Both parents non-UK born	790	10.9	409	12.2	199	9.3	182	10.4
Parents highest education								
No qualification	436	6.0	282	8.4	95	4.4	59	3.4
Other qualification	452	6.2	253	7.6	120	5.6	79	4.5
GCSE etc	1453	20.0	725	21.6	393	18.3	335	19.1
A-level etc	1443	19.9	668	19.9	439	20.4	336	19.2
Other high degree	1167	16.1	538	16.1	352	16.4	277	15.8
Degree	2302	31.7	885	26.4	750	34.9	667	38.1
Length of residency								
1 year or less	289	4.0	270	8.1	14	0.7	5	0.3
2–3 years	582	8.0	385	11.5	179	8.3	18	1.0
4–10 years	3134	43.2	1393	41.6	967	45.0	774	44.2
10 years or more	3248	44.8	1303	38.9	989	46.0	956	54.5
Parents’ mental well-being M(SD) [range]	7253	48.5(9.2) [3.0/71.0]	3351	48.0(9.4) [3.0/69.7]	2149	48.3(9.0) [7.3/71.0]	1753	48.1(8.8) [9.0/69.3]
At least one parent works	5973	82.4	2657	79.3	1773	82.5	1543	88.0
Household income								
Tertile 1	2456	33.9	1372	40.9	625	29.1	459	26.2
Tertile 2	2392	33.0	1095	32.7	715	33.3	582	33.2
Tertile 3	2405	33.2	884	26.4	809	37.7	712	40.6
**Neighbourhood level measures**								
Economically active M(SD) [range]	7253	7(1) [3/9]	3351	7(1) [3/9]	2149	7(1) [3/9]	1753	7(6) [4/8]
Townsend Index Deprivation M(SD) [range]	7253	5(2.2) [−2.6/9.2]	3351	6(2.3) [−2.6/9.2]	2149	4(2.2) [−2.5/9.2]	1753	4(2.2) [−2.6/9.2]

Source: UK Household Longitudinal Survey (2015) [waves 1, 3, and 5]. Linked adult and youth questionnaire with aggregated MSOA-level data from census 2011.

**Table 4:** Multilevel regression results of the association between deprivation and social capital on poor mental health and life satisfaction.

	Mental Health						Life Satisfaction					
	Model 3		Model 4		Model 5		Model 3		Model 4		Model 5	
	*B*	*SE*	*B*	*SE*	*B*	*SE*	*B*	*SE*	*B*	*SE*	*B*	*SE*
**Fixed Effects: Individual**												
Wave	−0.02	0.04	−0.03	0.04	−0.03	0.04	−0.00	0.01	−0.01	0.01	−0.01	0.01
Girls	−0.09	0.14	−0.09	0.14	−0.08	0.14	−0.10 ***	0.03	−0.11 ***	0.03	−0.11 ***	0.03
Cohorts (ref:1999)												
1998	−0.41+	0.21	−0.40+	0.21	−0.40+	0.21	−0.03	0.05	−0.03	0.05	−0.03	0.05
1997	−0.30	0.19	−0.25	0.19	−0.25	0.19	−0.13 **	0.04	−0.14 ***	0.04	−0.14 ***	0.04
1996	−0.36+	0.21	−0.36+	0.21	−0.35+	0.21	−0.23 ***	0.04	−0.22 ***	0.04	−0.23 ***	0.04
1995	−0.04	0.21	−0.03	0.21	−0.03	0.21	−0.34 ***	0.04	−0.35 ***	0.04	−0.35 ***	0.04
1994	0.06	0.22	0.07	0.22	0.07	0.22	−0.39 ***	0.05	−0.39 ***	0.05	−0.39 ***	0.05
Ethnicity (ref: white)												
Mixed	−0.58+	0.31	−0.64 *	0.31	−0.63 *	0.31	0.02	0.06	0.03	0.06	0.02	0.06
Asians	−1.08 ***	0.29	−1.12 ***	0.30	−1.03 ***	0.30	0.07	0.06	0.07	0.06	0.06	0.06
Blacks	−1.60 ***	0.32	−1.72 ***	0.33	−1.67 ***	0.33	0.10	0.07	0.12+	0.07	0.11	0.07
All other ethnicity	−0.73	0.80	−0.91	0.82	−0.94	0.82	0.14	0.17	0.19	0.17	0.18	0.17
Single parent household	0.02	0.20	−0.00	0.20	−0.01	0.20	−0.10 *	0.04	−0.08 *	0.04	−0.08 *	0.04
Parents nativity (ref:UK born)												
1 parent non-UK born	−0.53 *	0.27	−0.55*	0.27	−0.58 *	0.27	0.04	0.05	0.04	0.05	0.05	0.05
Both parents non-UK born	−0.82 **	0.30	−0.85**	0.31	−0.85 **	0.31	−0.02	0.06	−0.01	0.06	−0.01	0.06
Parents’ highest education (ref:No qualification)												
Other qualification	1.06 **	0.40	0.96*	0.40	0.90 *	0.40	−0.11	0.08	−0.08	0.08	0.90 *	0.40
GCSE or similar	0.34	0.33	0.30	0.33	0.25	0.33	−0.07	0.07	−0.06	0.07	0.25	0.33
A-level or similar	0.21	0.34	0.15	0.34	0.12	0.34	−0.07	0.07	−0.06	0.07	0.12	0.34
Other high degree	0.05	0.35	−0.01	0.35	−0.04	0.35	−0.04	0.07	−0.02	0.07	−0.04	0.35
Degree	−0.46	0.34	−0.51	0.34	−0.52	0.34	−0.04	0.07	−0.01	0.07	−0.52	0.34
Length of residency (ref:a year or less)												
2–3 years	−0.38	0.35	−0.44	0.36	−0.44	0.36	−0.11	0.08	−0.08	0.08	−0.07	0.08
4–10 years	−0.40	0.33	−0.45	0.34	−0.45	0.34	−0.07	0.07	−0.06	0.07	−0.05	0.07
10 years or more	−1.06 **	0.34	−1.13 **	0.34	−1.12 **	0.34	−0.07	0.07	−0.06	0.07	−0.05	0.07
Parents’ mental well-being	−0.06 ***	0.01	−0.06 ***	0.01	−0.06 ***	0.01	−0.04	0.07	−0.02	0.07	−0.02	0.07
At least one parent works	−0.58 **	0.20	−0.54 **	0.20	−0.54 **	0.20	−0.04	0.07	−0.01	0.07	−0.00	0.07
Household income (ref:tertile 1)												
Tertile 2	0.32+	0.16	0.33 *	0.16	0.33 *	0.16	0.01	0.08	0.01	0.08	0.01	0.08
Tertile 3	0.06	0.19	0.08	0.19	0.10	0.19	−0.01	0.07	0.01	0.07	0.01	0.07
**Fixed Effects: Neighbourhood**							0.04	0.07	0.06	0.07	0.06	0.07
Economically active	3.00+	1.59	2.11	1.63	1.73	1.63	−0.95 **	0.31	−0.80 *	0.32	−0.82 **	0.32
Townsend Index Deprivation	0.13 **	0.05	0.08	0.06	0.12	0.14	−0.02 *	0.01	−0.02	0.01	−0.06 *	0.03
Not worried about crime			−0.74 *	0.35	−0.63+	0.36			0.19 **	0.07	0.17 *	0.07
Not worried about crime*deprivation					−0.11	0.17					0.04	0.03
Quality of facilities & amenities			0.42	0.60	0.63	0.60			−0.07	0.12	−0.08	0.12
Quality of facilities & amenities*deprivation					0.30	0.32					0.00	0.06
Civic engagement			−0.71+	0.37	−0.73+	0.38			0.03	0.07	0.02	0.07
Civic engagement*deprivation					0.28	0.17					0.01	0.03
Friendship networks			−0.87 **	0.28	−1.07 ***	0.29			0.14*	0.06	0.19 ***	0.06
Friendship networks*deprivation					0.43 ***	0.13					−0.08 ***	0.02
Trust and cooperative norms			−0.24	0.59	−0.11	0.61			0.12	0.12	0.10	0.12
Trust & cooperative norms*deprivation					−0.31	0.26					0.04	0.05
Social Cohesion			−0.14	1.11	−0.07	1.17			−0.16	0.22	−0.21	0.23
Social cohesion*deprivation					0.18	0.50					0.07	0.10
Constant	13.18 ***	1.24	14.49 ***	1.30	14.84 ***	1.31	6.25 ***	0.25	6.03 ***	0.26	6.06 ***	0.26
**Variance components: neighbourhoods**												
Slope	0.41 **	0.12	0.40 **	0.12	0.40 **	0.12	0.10 ***	0.02	0.10 ***	0.02	0.10 ***	0.02
Between neighbourhoods	1.85 ***	0.27	1.81 ***	0.28	1.77 ***	0.28	0.40 ***	0.06	0.39 ***	0.06	0.39 ***	0.06
Intercept/slope covariance	−0.51 *	0.19	−0.53 *	0.19	−0.54 *	0.19	−0.72 ***	0.11	−0.74 ***	0.10	−0.76 ***	0.10
**Variance components: youth**												
Slope	0.80+	0.11	0.81	0.11	0.81	0.11	0.15 ***	0.03	0.14 ***	0.03	0.15 ***	0.03
within neighbourhood/between youths	4.26 ***	0.21	4.29 ***	0.21	4.29 ***	0.21	0.76 ***	0.06	0.76 ***	0.06	0.76 ***	0.06
Intercept/slope covariance	−0.48 ***	0.07	−0.49 ***	0.07	−0.49 ***	0.07	−0.62 ***	0.08	−0.63 ***	0.08	−0.63 ***	0.08
within youth/wave	3.39 ***	0.08	3.38 ***	0.08	3.38 ***	0.08	0.85 ***	0.02	0.85 ***	0.02	0.85 ***	0.02
N	7253		7157		7157		7253		7157		7157	

Note: Significant at + p < 0.10,* p < 0.05, ** p < 0.01, *** p < 0.001. Source: UK Household Longitudinal Survey (2015) [waves 1, 3, and 5]. Linked adult and youth questionnaire with aggregated MSOA-level data from census 2011.
